# Supplementary material for: Viscosity-dependent control of protein synthesis and degradation
Source: Nat Commun. 2024 Mar 8;15:2149. doi: 10.1038/s41467-024-46447-w (PMC10923802; doi:10.1038/s41467-024-46447-w)
Supplement: Supplementary file 1 — Supplementary Information [file 41467_2024_46447_MOESM1_ESM.pdf]

Supplementary Materials for

Viscosity-dependent control of protein synthesis and degradation

Yuping Chen, Jo-Hsi Huang, Connie Phong, and James E. Ferrell, Jr.

Correspondence to: cyp7cn@gmail.com (Y.C.); james.ferrell@stanford.edu (J.E.F.)

This PDF file includes:

Supplementary Text

Supplementary References (*1-4*)

Supplementary Figures and Captions (*1-4*)

Supplementary Text

Models for the effect of diffusion on reaction

We begin by deriving a simple model to account for the observed rates of protein translation and degradation as a function of cytosolic protein concentration assuming a mass action kinetics. We assume that the rate determining reaction for each process is a mass action bimolecular reaction:

$$V = \frac{dP}{dt} = kE \cdot S \tag{1}$$

where V is the rate of the reaction, P is the product of the reaction, E is the enzyme, S the substrate, and P the product of the reaction. Alternatively, we could assume a two-step, saturable mechanism (see below). The enzyme and substrate concentrations are linearly proportional to the relative cytoplasmic concentration ϕ. We can therefore write:

$$E[\phi] = \phi E[1] \tag{2}$$

$$S[\phi] = \phi S[1], \tag{3}$$

where $E[\phi]$ and $S[\phi]$ are the enzyme and substrate concentrations at a relative cytoplasmic concentration of ϕ, and $E[1]$ and $S[1]$ are the enzyme and substrate concentrations at a relative cytoplasmic concentration 1x. Substituting into Eq. S1 yields:

$$V = k\phi^2 E[1]S[1]. \tag{4}$$

Next we consider the dependence of the rate constant on the cytoplasmic protein concentration. From the Smoluchowski equation[1], the collision rate and the association rate constant k are proportional to the sum of the diffusion coefficients of E and S, and from Phillies's law (Eq. 1 in the main text) [2,3], we take the diffusion coefficients to be negative exponential functions of the cytoplasmic protein concentration:

$$k[\phi] \propto D_E[\phi] + D_S[\phi] = D_E[0]e^{-\mu_E \phi} + D_S[0]e^{-\mu_S \phi}, \tag{5}$$

where $D_E[0]$ and $D_S[0]$ are the diffusion coefficients of the enzyme and the substrate at a cytoplasmic concentration of 0 (i.e. in filtrate), and the μ's are scaling factors. For the special case where either one diffusion coefficient is much smaller than the other, or the scale factors are equal, we can simplify this to:

$$k[\phi] = k[0]e^{-\mu\phi}. \tag{6}$$

Note that it is a bit awkward to consider a value of k at a relative cytoplasmic concentration of 0, since the reacting species are cytoplasmic macromolecules that are absent from the filtrate. We can avoid this by instead writing:

$$k[\phi] = k[1]e^{-\mu\,(\phi-1)}. \tag{7}$$

It follows that:

$$V = k[1]E[1]S[1]\left(\phi^2 e^{-\mu\,(\phi-1)}\right). \tag{8}$$

Finally, we can make use of the fact that the scaling factor μ is linearly proportional to the size of the diffusing particle d_p, and write an expression that explicitly acknowledges particle size:

$$V = k[1]E[1]S[1]\left(\phi^2 e^{-ad_p\,(\phi-1)}\right). \tag{9}$$

where a is a new scaling factor that relates d_p to μ. Eq. S9 describes how the rate of a mass action, bimolecular reaction whose reactants obey Phillies's law would be expected to vary with the cytoplasmic macromolecule concentration and molecular diameter. If we measure rates in arbitrary units with the rate at $\phi = 1$ taken as 1, then:

$$V = \phi^2 e^{-ad_p\,(\phi-1)}. \tag{10}$$

Note that we have an experimental estimate for a (which, from Fig. 4f is 0.018 nm^{-1}), leaving only one adjustable parameter, d_p, the macromolecular diameter. Eq. S10 defines a biphasic, non-monotonic curve (Fig. 5a), and the larger the assumed macromolecular diameter, the further to the left the curve's maximum lies (Fig. 5a). For a given value of d_p, the optimal cytoplasmic concentration is given by:

$$\phi_{optimal} = \frac{2}{ad_p}. \tag{11}$$

This relationship is plotted in Fig. 5b.

Alternatively, we can assume that the rate determining reaction for each process is a two-step enzymatic reaction:

$$E + S \underset{k_{-1}}{\overset{k_1}{\rightleftharpoons}} C \xrightarrow{k_2} E + P$$

where E is the enzyme, S the substrate, C the enzyme-substrate complex, and P the product of the reaction. If we assume that the system is in steady state, with $\frac{dC}{dt} = 0$, and that the substrate concentration is much higher than the enzyme concentration, then the rate of this process is described by the Michaelis-Menten equation:

$$V = \frac{dP}{dt} = \frac{k_2 E_{tot} S}{\frac{k_{-1}+k_2}{k_1} + S}, \tag{12}$$

where $E_{tot} = E + C$.

Next we want to add cytoplasmic concentration dependence to the terms on the right-hand side of Eq. S12. The enzyme and substrate concentrations are linearly proportional to the relative cytoplasmic concentration ϕ. We can therefore write:

$$E_{tot}[\phi] = \phi E_{tot}[1], \tag{13}$$

$$S[\phi] = \phi S[1]. \tag{14}$$

Substituting into Eq. S12 yields:

$$V = \frac{k_2 \phi^2 E_{tot}[1] S[1]}{\frac{k_{-1}+k_2}{k_1} + \phi S[1]}. \tag{15}$$

Again, from the Smoluchowski equation [1] and Phillies's law [2,3], we take the diffusion coefficients to be negative exponential functions of the cytoplasmic protein concentration:

$$k_1[\phi] \propto D_E[\phi] + D_s[\phi] = D_E[0] e^{-\mu_E \phi} + D_S[0] e^{-\mu_S \phi}. \tag{16}$$

For the special case where either one diffusion coefficient is much smaller than the other, or the scale factors are equal, we can simplify this to:

$$k_1[\phi] = k_1[0] e^{-\mu \phi}. \tag{17}$$

Similarly, we can rewrite Eq. S17 as:

$$k_1[\phi] = k_1[1]e^{-\mu\,(\phi-1)}. \tag{18}$$

Some models of protein complex dissociation assume that the rate of dissociation is directly proportional to the sum of the diffusion coefficients of the proteins[4]. Therefore we assumed that $k_{-1}$is directly proportional to the diffusion coefficient, which varies with the cytoplasmic concentration according to Phillie's law, and that k_2, the rate constant for the catalytic step, is independent of the cytoplasmic concentration. It follows that:

$$V = \frac{k_2\phi^2 E_{tot}[1]S[1]}{\frac{k_{-1}[1]e^{-\mu\,(\phi-1)}+k_2}{k_1[1]e^{-\mu\,(\phi-1)}}+\phi S[1]}. \tag{19}$$

Finally, since the scaling factor μ is linearly proportional to the size of the diffusing particle d_p, we can write an expression that explicitly acknowledges particle size:

$$V = \frac{k_2\phi^2 E_{tot}[1]S[1]}{\frac{k_{-1}[1]e^{-ad_p\,(\phi-1)}+k_2}{k_1[1]e^{-ad_p\,(\phi-1)}}+\phi S[1]}, \tag{20}$$

where a is a new scaling factor that relates d_p to μ. Eq. S20 describes how the rate of a Michaelis-Menten reaction whose reactants obey Phillies's law and the Smoluchowski equation would be expected to vary with the cytoplasmic macromolecule concentration and molecular diameter.

We do not have experimental estimates for most of the parameters in Eq. S20 (except for a, which, from Fig. 4f is 0.018 nm^{-1}) for either translation or protein degradation. However, we can get a feel for Eq. S20 by arbitrarily assuming some parameter values ($k_1[1] = 0.01$; $E_{tot}[1] = S[1] = k_{-1}[1] = k_2[1] = 1$) and plotting V as a function of ϕ for macromolecules of different assumed macromolecular diameters. The equation defines a biphasic, non-monotonic curve (Supplementary Fig. 4a), and the larger the assumed macromolecular radius, the further to the left the curve's maximum lies. The same was true for the mass action model (Eq. 11 and Fig. 5). The ratio between k_{-1} and k_2, which determines whether the system is reaction-controlled or diffusion-controlled, also affects the optimal value of ϕ; the more diffusion-controlled the system is, the further to the left the optimum is (Supplementary Fig. 4b).

Supplementary Figures

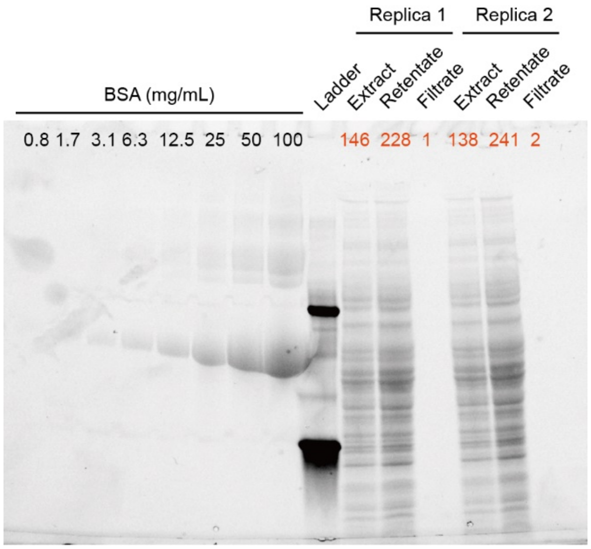

Supplementary Fig. 1: Trihalo compound-stained PAGE gel of proteins from 1x extract, filtrate, and 2x retentate.

a representative trihalo compound-stained PAGE gel showing a BSA standard and two biological replicates of extract, retentate, and filtrate. The estimated protein concentrations in mg/mL (using the BSA standard as a reference) for the extract, retentate, and filtrate samples are shown in orange. However, it should be noted that the trihalo compound used in staining the gel depends on tryptophan residues to fluoresce. Since BSA has only 0.3% tryptophan residues (compared to 1% in proteins overall), the protein concentrations in the extract, retentate, and filtrate samples are likely overestimated. By Bradford assay the typical protein concentration of extracts was 50-70 mg/mL.

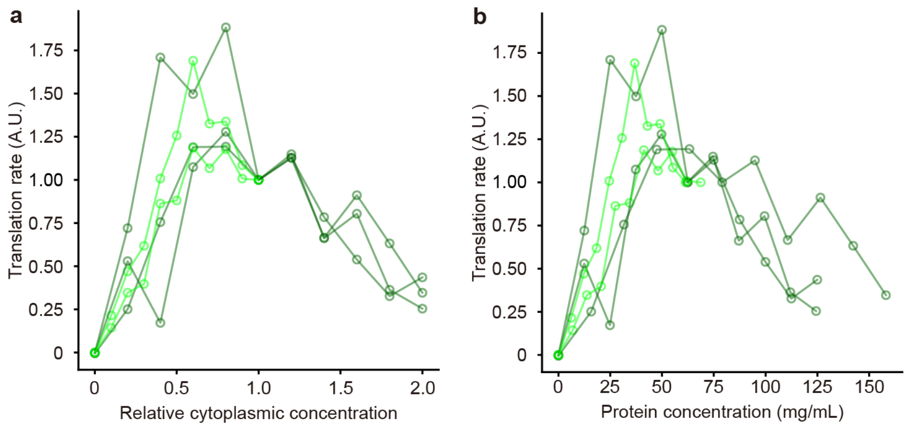

Supplementary Fig. 2: Using nominal vs. measured protein concentrations to compare translation rates in different experiments.

a Translation rate as a function of nominal cytoplasmic concentration. These are the directly-measured data from experiments where the eGFP mRNA concentration was kept constant and the translation machinery was proportional to the cytoplasmic concentration. Data points from same experiment are connected. Data are normalized relative to the translation rates at a cytoplasmic concentration of 1x. Relative cytoplasmic concentration are assumed to be 1.0 for 1x extract and 2.0 for the 2x retentate.

b Translation rate as a function of measured protein concentration. These are the directly-measured data from experiments where the eGFP mRNA concentration was kept constant and the translation machinery was proportional to the cytoplasmic concentration. Data points from same experiment are connected. Data are normalized relative to the translation rates at a cytoplasmic concentration of 1x. Protein concentrations were measured for the starting extract and the retentate, instead of assuming that they were 1x and 2x. Protein concentrations for the dilutions were calculated from these the respective starting material. Note that the experiment-to-experiment variation is similar regardless of whether nominal or measured protein concentrations are used.

Source data are provided as a Source Data file.

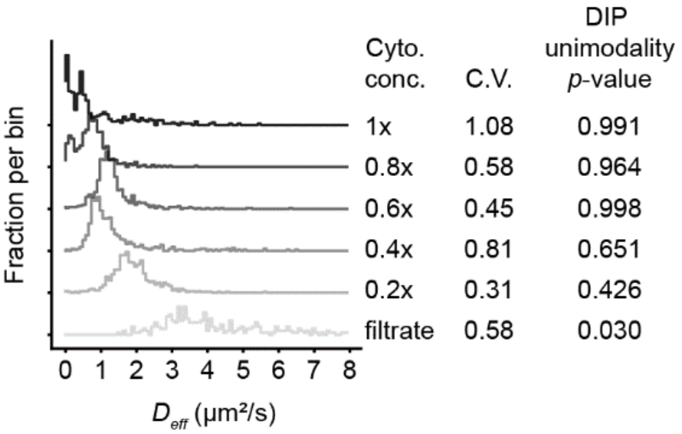

Supplementary Fig. 3: Probability density of effective diffusion in various cytoplasmic concentrations.

Histograms of the effective diffusion coefficient for 100 nm PEGylated polystyrene beads are plotted for extracts with various cytoplasmic dilutions, with offsets. The cytoplasmic concentration, coefficients of variation (C.V.), and *p*-values for the DIP test of unimodality are shown next to each curve.

Source data are provided as a Source Data file.

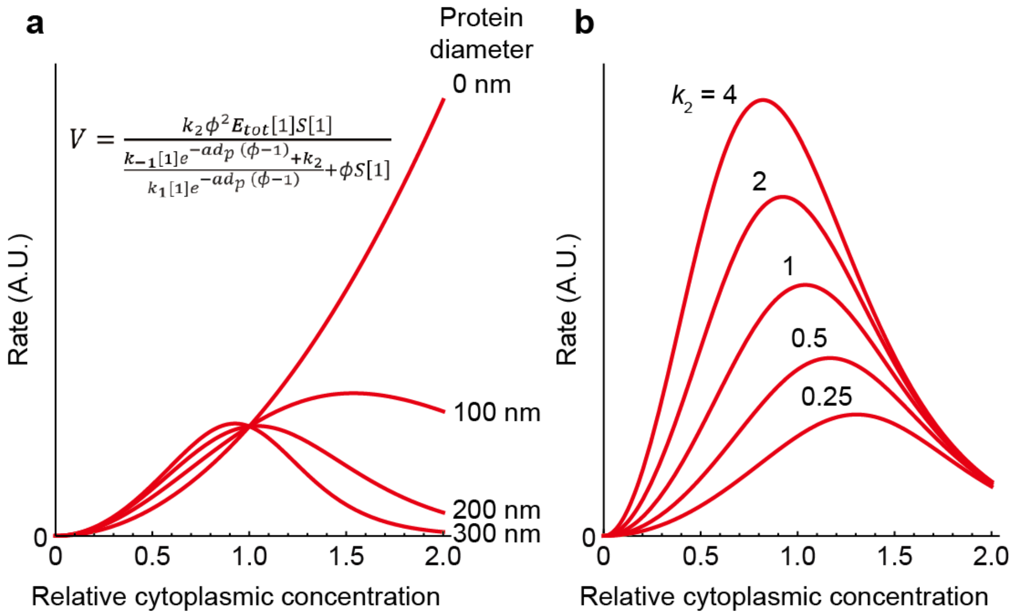

Supplementary Fig. 4: The effect of cytoplasmic concentration on translation and protein degradation in a Michaelis-Menten model.

a Plot of Eq. S20, which relates a bimolecular reaction rate to the relative cytoplasmic concentration, for various sizes of proteins, assuming that Michaelis-Menten kinetics are relevant. We assumed $a = 0.018$ nm^{-1} (from Fig. 4f) and arbitrarily chose the following values for the parameters: $k_1[1] = 0.01$ (so that the reaction was not close to saturation) and $E_{tot}[1] = S[1] = k_{-1}[1] = k_2[1] = 1$ (so that the reaction was neither close to the reaction limit nor to the diffusion limit).

b Plot of Eq. S20 for various assumed values of k_2, as indicated. Larger values make the system more diffusion-controlled and smaller values make it more reaction-controlled. We again assumed $a = 0.018$ nm^{-1} and arbitrarily chose the following values for the parameters: $k_1[1] = 0.01$, $E_{tot}[1] = S[1] = k_{-1}[1]$, and the protein size to be 100 nm. The *y*-axis scales are different in panels **(a)** and **(b)**.

Supplementary References

1. Smoluchowski, M. Mathematical theory of the kinetics of the coagulation of colloidal solutions. Zeitschrift für Physikalische Chemie 92, 129-168 (1917).
2. Phillies, G. D. J. Quantitative prediction of .alpha. in the scaling law for self-diffusion. Macromolecules 21, 3101-3106 (2002). https://doi.org:10.1021/ma00188a031
3. Phillies, G. D. Universal scaling equation for self-diffusion by macromolecules in solution. Macromolecules 19, 2367-2376 (1986).
4. DeLisi, C. The biophysics of ligand-receptor interactions. Q Rev Biophys 13, 201-230 (1980). https://doi.org:10.1017/s0033583500001657
