# Supplementary material for: Viscosity-dependent control of protein synthesis and degradation
Source: Nat Commun. 2024 Mar 8;15:2149. doi: 10.1038/s41467-024-46447-w (PMC10923802; doi:10.1038/s41467-024-46447-w)
Supplement: Supplementary file 3 — Description of Additional Supplementary Files [file 41467_2024_46447_MOESM3_ESM.pdf]

Description of Additional Supplementary Files:

Supplementary Movie 1: Effects of cytoplasmic concentration on self-organization and cycling in dilutions from 1x extract. SiR-tubulin staining (left) and SiR-tubulin fluorescence intensity as a function of time (right) in an extract after various dilutions. The same data are shown in Fig. 1d. The starting material was a 1x extract, diluted with various proportions of filtrate and imaged in a 96-well plate under mineral oil. All fields are shown at equal exposure. The fluorescence intensities shown on the right were quantified from the center 1/9 of the wells and subsequently rescaled using the maximum and minimum values to enhance the variability in the intensities, particularly in cases of low cytoplasmic concentration.

Supplementary Movie 2: Effects of cytoplasmic concentration on self-organization and cycling in dilutions from 2x retentate. SiR-tubulin staining (left) and SiR-tubulin fluorescence intensity as a function of time (right) in an extract after various dilutions. The data were collected as in Supplementary Movie 1 except that the starting material was a 2x retentate. The same data are shown in Fig. 1e.

Supplementary Movie 3: Cell-like compartment formation and cell cycle oscillation in 0.2x and 0.3x cytoplasm. SiR-tubulin staining was conducted on a 0.2x extract (left) and a 0.3x extract (right) as shown in Supplementary Movie1 and Fig. 1d. The fluorescence intensities were rescaled to improve the visibility of the SiR-tubulin staining, and are displayed at a higher magnification.
