# Supplementary figures and images for: Viscosity-dependent control of protein synthesis and degradation
Source: Nat Commun. 2024 Mar 8;15:2149. doi: 10.1038/s41467-024-46447-w (PMC10923802; doi:10.1038/s41467-024-46447-w)

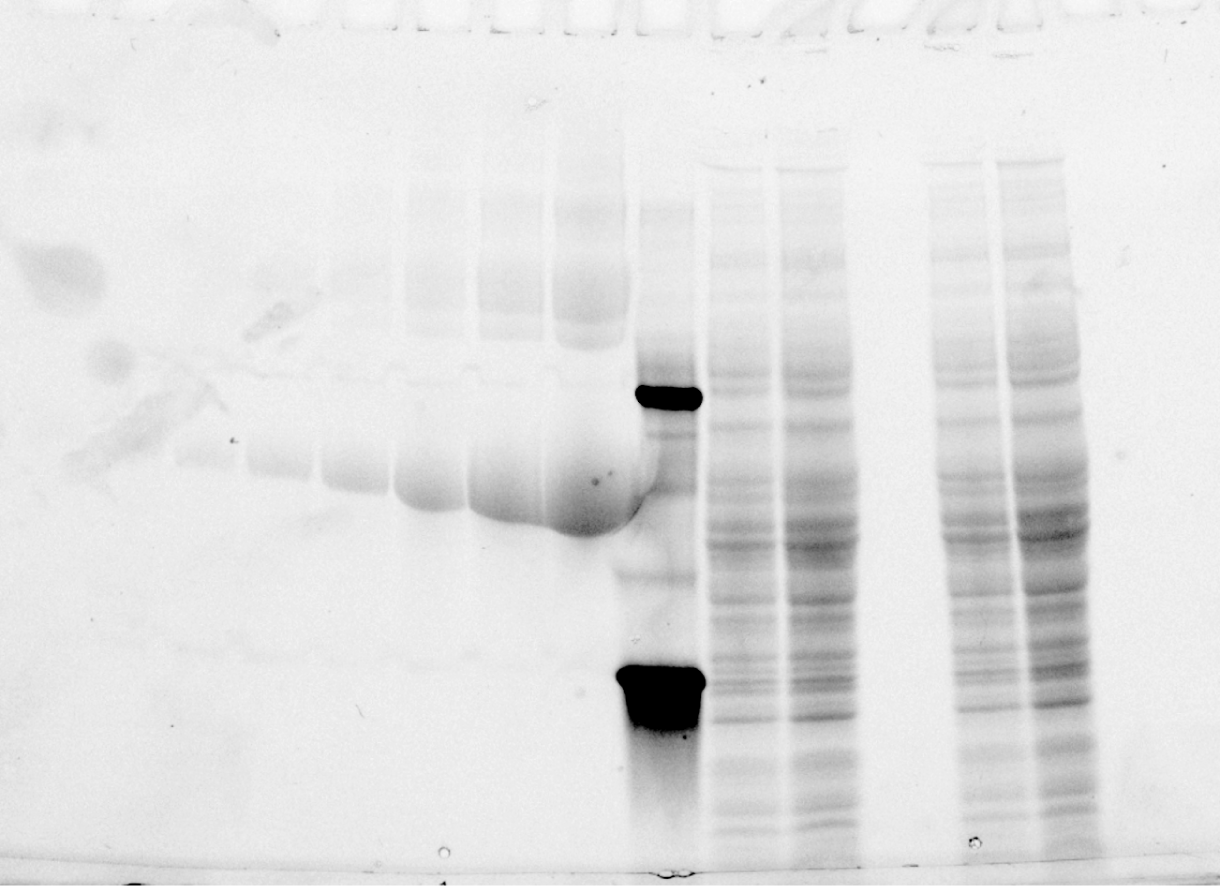

Supplement: Supplementary file 8 — Source Data [file 41467_2024_46447_MOESM8_ESM.zip › Source Data - Figure S1.tif]
